# *Atmin* modulates *Pkhd1* expression and may mediate Autosomal Recessive Polycystic Kidney Disease (ARPKD) through altered non-canonical Wnt/Planar Cell Polarity (PCP) signalling

**DOI:** 10.1016/j.bbadis.2018.11.003

**Published:** 2019-02-01

**Authors:** Taylor Richards, Kavindiya Modarage, Charlotte Dean, Aidan McCarthy-Boxer, Helen Hilton, Chris Esapa, Jill Norman, Patricia Wilson, Paraskevi Goggolidou

**Affiliations:** a School of Biomedical Science and Physiology, Faculty of Science and Engineering, University of Wolverhampton, Wulfruna Street, Wolverhampton WV1 1LY, UK; b National Heart and Lung Institute, Imperial College, South Kensington Campus, London SW7 2AZ, UK; c MRC Harwell Institute, Mammalian Genetics Unit, Harwell Campus, Oxfordshire OX11 0RD, UK; d Centre for Nephrology, UCL Medical School, Royal Free Campus, Rowland Hill, London NW3 2PF, UK

**Keywords:** ADPKD, Autosomal Dominant Polycystic Kidney Disease, ARPKD, Autosomal Recessive Polycystic Kidney Disease, CT, collecting tubule, Dvl, Dishevelled, Fz, Frizzled, mIMCD3, mouse inner medullary collecting duct 3, PC, polycystin, PCP, Planar Cell Polarity, ATMIN, PKHD1, Wnt signalling, ARPKD

## Abstract

Autosomal Recessive Polycystic Kidney Disease (ARPKD) is a genetic disorder with an incidence of ~1:20,000 that manifests in a wide range of renal and liver disease severity in human patients and can lead to perinatal mortality. ARPKD is caused by mutations in *PKHD1*, which encodes the large membrane protein, Fibrocystin, required for normal branching morphogenesis of the ureteric bud during embryonic renal development. The variation in ARPKD phenotype suggests that in addition to *PKHD1* mutations, other genes may play a role, acting as modifiers of disease severity. One such pathway involves non-canonical Wnt/Planar Cell Polarity (PCP) signalling that has been associated with other cystic kidney diseases, but has not been investigated in ARPKD. Analysis of the *Atmin*^*Gpg6*^ mouse showed kidney, liver and lung abnormalities, suggesting it as a novel mouse tool for the study of ARPKD. Further, modulation of *Atmin* affected *Pkhd1* mRNA levels, altered non-canonical Wnt/PCP signalling and impacted cellular proliferation and adhesion, although Atmin does not bind directly to the C-terminus of Fibrocystin. Differences in ATMIN and VANGL2 expression were observed between normal human paediatric kidneys and age-matched ARPKD kidneys. Significant increases in *ATMIN*, *WNT5A*, *VANGL2* and *SCRIBBLE* were seen in human ARPKD versus normal kidneys; no substantial differences were seen in *DAAM2* or *NPHP2*. A striking increase in E-cadherin was also detected in ARPKD kidneys. This work indicates a novel role for non-canonical Wnt/PCP signalling in ARPKD and suggests *ATMIN* as a modulator of *PKHD1*.

## Highlights

• The *Atmin*^*Gpg6*^ mouse displays kidney, liver and lung defects.
• *Atmin* modulates *Pkhd1* expression and affects cellular proliferation and adhesion in mIMCD3 cells.
• Altered Wnt signalling is observed in *Atmin/Pkhd1* knockdowns and in ARPKD kidneys.

## Graphical abstract

**Figure:**
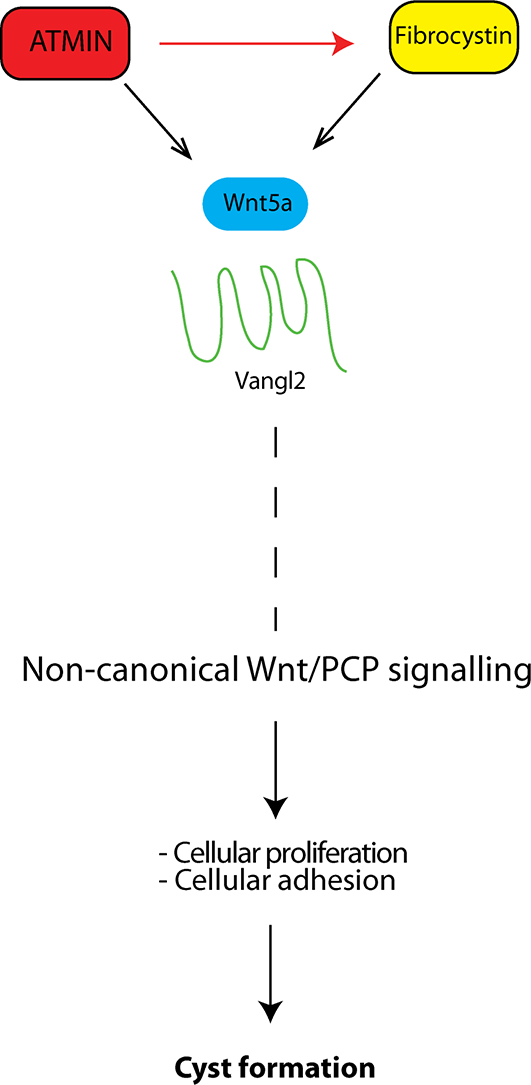

## 1 Introduction

Autosomal Recessive Polycystic Kidney Disease (ARPKD) is a rare hereditary disorder, affecting 1:20,000 to 1:40,000 individuals, mostly foetuses and infants [1] and is a common cause of perinatal death [2]. It manifests as extreme bilateral enlargement of cystic kidneys in utero, associated with hepatic ductal plate abnormalities and pulmonary hypoplasia [3,4]. In those patients who survive the perinatal period, the majority will require renal replacement therapy (dialysis/transplantation) within the first decade [5]. Recently, however, ARPKD patients have been diagnosed in their 30s with relatively mild renal insufficiency [6]. Besides the typical renal manifestations associated with ARPKD, a significant percentage of ARPKD patients manifest extra-renal phenotypes, such as liver fibrosis and dilated bile ducts (Caroli's syndrome) [7], suggesting a previously unrecognised, wide spectrum of disease severity.

ARPKD is caused by mutations in *PKHD1*, the gene encoding Fibrocystin. Fibrocystin is a membrane-associated receptor-like protein of 447 kDa, composed of a large, modular extracellular N-terminal domain, a single transmembrane domain and a short intracellular C-terminal region [8]. Many different mutations in *PKHD1* have been reported throughout the whole gene and the combination of mutations determines the phenotype of ARPKD patients. Patients with two truncating mutations have a lethal phenotype, whereas the presence of at least one missense change can be compatible with life, indicating that many missense mutations are hypomorphic alleles that generate partially functional protein [9]. Modifier genes are also believed to play a role in the range of disease severity observed in ARPKD [4,[10], [11], [12]].

Fibrocystin is required for normal branching morphogenesis of the ureteric bud during embryonic renal development [8,13]. In ARPKD kidneys, cystic dilation is restricted to the ureteric bud-derived collecting tubules (CTs) and is associated with increased epithelial cell proliferation and luminal fluid secretion [14], as well as abnormalities in apoptosis [15,16], epithelial cell polarity [17] and cell-matrix interactions [18]. Fibrocystin is also localised in primary cilia protruding from the apical surface of CT cells [19,20], together with Polycystins (PC)-1 and -2 [21], the protein products of the Autosomal Dominant (AD)PKD-causative genes *PKD1* and *PKD*-*2* [3,4]. In the kidney, primary cilia are non-motile sensory organelles that act as signal transducers involved in cell signalling pathways [22]. Both canonical Wnt and non-canonical Wnt/PCP pathways are relevant to ciliary signalling and kidney development [23]. Loss of Fibrocystin function causes shorter cilia in the bile ducts of a mouse model with no functional Fibrocystin [24].

Canonical Wnt signalling is initiated when Wnt ligands bind to Frizzled (Fz) receptors in the presence of LRP5 or 6 [25]. This results in Dishevelled (Dvl) activation and stabilisation of β-catenin, which then translocates to the nucleus and initiates transcriptional activation of Wnt target genes. The other key downstream Wnt signalling pathway in the kidney is the non-canonical Wnt/PCP pathway. The binding of non-canonical Wnt ligands here results in the recruitment of Fz and Dvl to the membrane, culminating in cytoskeletal rearrangements that affect cellular organisation, shape and migration. In the kidney, interaction of Inversin with Dvl is hypothesised to trigger non-canonical Wnt/PCP pathway activation [26]. Many core and effector PCP proteins have been implicated in kidney development and disease and one of these is Atmin, a transcription factor with diverse roles in DNA damage repair and ciliogenesis [[27], [28], [29]]. Kidney development was demonstrated to be greatly impaired in *Atmin* deficient mice, with homozygous mutant kidneys displaying reduced numbers of differentiated ureteric buds and renal vesicles [30]. Loss of Atmin resulted in changes in expression of non-canonical Wnt components (*Wnt9b*, *Wnt11*, Vangl2, *Daam2*) that affected oriented cell division and the cytoskeletal organisation in the renal epithelial cells of the kidney.

The significant role of Wnt signalling is gaining recognition in cystic renal disease. Disrupted Wnt signalling components, including Wnt9b, Fat4, Vangl2 and Inversin are associated with polycystic kidney disease [[31], [32], [33], [34], [35], [36]] and frizzled-related-protein-4 is upregulated in human ADPKD and in ADPKD mouse models [37]. Furthermore, DKK3, a β-catenin antagonist, is a potential modifier of ADPKD [38] and Wnt ligands bind to the *PKD1*-encoded PC-1 extracellular domain and activate the PC-1/PC-2 channel [39]. While defective Wnt signalling has been implicated in ADPKD and mis-oriented cell division was detected in *Pck* rats [40], the role of non-canonical Wnt/PCP signalling has not been investigated in detail in human ARPKD. Hence an examination of novel Wnt pathways implicated in ARPKD was conducted in mice, cell lines and human kidneys, providing a unified approach into understanding paediatric polycystic kidney disease mechanisms.

## 2 Materials and methods

### 2.1 Mice

The ENU-derived *Atmin*^*Gpg6*^ mice were identified in an ENU mutagenesis screen at MRC Harwell, as previously described [27,41]. *Gpg6* mice show a T to A transversion in exon 3 of *Atmin*, correlating with the third Zinc Finger. This results in a cysteine to serine substitution in the fourth canonical residue. Genotyping was carried out using pyrosequencing assays to directly amplify the mutations; wildtype littermates were used as controls (primers available on request). Work was conducted under Home Office project licence number 30/3286.

### 2.2 Antibodies

Western blotting was carried out using 40 μg of whole tissue lysate per lane with the E-cadherin antibody (1:1000, 3195, Cell signalling), β-catenin antibody (1:1000, 9582, Cell signalling), non-phospho active β-catenin antibody (1:1000, 8814S, Cell signalling), GAPDH (1:1000, ab8245, Abcam) and β-actin antibody (1:5000, A5316, Sigma). For the *Atmin* siRNA-mediated knockdown immunocytochemistry, E-cadherin (1:500, 3195, Cell signalling) was used.

### 2.3 Cell culture and siRNA knockdowns

Mouse inner medullary collecting duct (mIMCD3) cells (gift from D. Norris, MRC Harwell) were grown in DMEM/F12 (Gibco) media supplemented with 2% fetal bovine serum (Life Technologies) and penicillin-streptomycin (Life Technologies) in 1% collagen-coated 6-well plates (VWR). mIMCD3 cells were transfected with Lipofectamine RNAimax transfection reagent (Thermo Fisher), using siRNA to knock down *Atmin*, *Pkhd1* or *Pkhd1* and *Atmin*, according to the manufacturer's instructions (Thermo Fisher); scrambled siRNA was used as a control (Thermo Fisher). For each experiment, at least three biological samples per group were used and for each sample, three technical replicates were performed.

### 2.4 Quantitative RT-PCR analysis

To measure gene expression levels, RNA was extracted using the RNeasy mini kit as per manufacturer's instructions (QIAGEN). 1 μg of RNA was isolated per sample and cDNA was prepared for qRT–PCR using the High Capacity cDNA Reverse transcription kit (ABI). qRT-PCR was performed for: *ATMIN*, *DAAM2*, *NPHP2*, *WNT5A*, *VANGL2*, *SCRIBBLE*, *β*-*CATENIN*, *Atmin*, *Pkhd1*, *Scribble*, *Wnt5a*, *Vangl2*, *Nphp2*, *Axin2*. All assays were provided by ABI and were run on the ABI ViiA 7 Real-Time PCR system. Alterations in gene expression were expressed relative to the mean intensity in control (normal human kidney or scrambled control) over B2M and β-actin expression respectively, which was given a standardized value of 1. Negative controls without cDNA template were included. All qRT–PCR assays were performed in triplicate. Primer details are available upon request.

### 2.5 Proliferation and apoptotic analysis

Proliferation and apoptotic indices were calculated by counting the numbers of phospho-histone-H3 (1:500, 06–570, Millipore), or cleaved caspase 3 (1:500, 9661, Cell signalling)-positive cells, respectively, in 10 comparable fields per mouse kidney or gene knockdown, as a percentage of DAPI-stained nuclei (*n* = 4 biological samples per genotype or group). Immunofluorescence analysis was carried out using a Leica DM2500 optical microscope with the Leica applications suite software. Adobe Photoshop CS7 software was used to compose and analyse the images.

### 2.6 Cell adhesion analysis

Post siRNA knockdown, 50,000 mIMCD3 cells were grown as described above, washed twice in PBS, fixed for 10 min in 4% PFA and then stained for 10 min with 0.1% crystal violet solution. Stained cells were washed off with running water until the run-off became clear, left to dry overnight and then solubilised in 100% methanol. The solubilised crystal violet was read in a 570 nm wavelength spectrophotometer (Thermo Labsystems Multiskan MS plate reader). Results were obtained by subtracting background absorbance, were deducted from control sample and multiplied by 100% for percentage adherence (n = 4 biological samples per group).

### 2.7 Overexpression studies, immunoprecipitation and Western blot analysis of transfected cells

mIMCD3 cells were transfected with ATMIN-MYC cDNA (gift from D. Norris, MRC Harwell) using *jetPRIME* transfection reagent (Polyplus). After 48 h, RNA was extracted using the RNeasy mini kit as per manufacturer's instructions (QIAGEN).

Human embryonic kidney HEK293T cells were transfected with either ATMIN-MYC cDNA (gift from D. Norris, MRC Harwell), PKHD1-GFP cDNA (Plasmid no. 45560, Addgene) or both, using *jetPRIME* transfection reagent (Polyplus). After 48 h, cells were lysed using lysis buffer (50 mM Tris pH 8, 150 mM NaCl, 1% NP-40 and EDTA-free protease (Roche) and PhosSTOP phosphatase (Roche) inhibitors) and centrifuged to remove insoluble debris. Immunoprecipitation from cleared lysates was performed using rabbit-anti-MYC (C3956, Sigma-Aldrich) and mouse-anti-GFP (11814460001, Roche) antibodies, respectively overnight at 4 °C. Primary antibodies were captured using Protein G sepharose (Sigma-Aldrich) for 2 h at 4 °C. Immunocomplexes were then washed using lysis buffer and eluted in electrophoresis LDS loading buffer (Invitrogen).

Cell lysates and immunoprecipitates were separated by electrophoresis using NuPAGE gel (Invitrogen) and transferred to nitrocellulose membranes using the iBlot transfer system (Invitrogen). Membranes were blocked for 1 h in blocking solution (5% skimmed milk in PBS, 0.1% Tween 20 (PBST)) and then incubated in primary antibody (rabbit-anti-MYC (Sigma Aldrich) or rabbit-anti-GFP (in-house)) diluted in blocking solution overnight at 4 °C. After extensive washing in PBST, membranes were incubated for 1 h in the corresponding secondary antibody (goat-anti-rabbit-HRP (A6154, Sigma Aldrich) for MYC blot and EasyBlot anti-rabbit-HRP (GTX221666-01, Genetex) for GFP blot) diluted in blocking solution and washed as above. Western blots were visualised by ECL.

### 2.8 Human tissue

Newborn to 18 years of age ARPKD and age-matched normal human kidney samples were procured in a sterile fashion either immediately post-mortem or from the operating room, at time of nephrectomy prior to transplant and anonymised at source by National Institute's of Health (NIH) ethically-approved National Disease Research Interchange (NDRI, Philadelphia, USA) with full Institutional Review Board (IRB)/NIH approval in USA and in UK with UCL ethical approval (Number 05/Q0508/6), processed and stored in the PKD Charity UK-sponsored Bioresource Bank. No samples had been subjected to warm ischaemia and all were routinely validated by a trained in-house pathologist.

### 2.9 Histology and immunostaining

Kidneys were fixed in 4% paraformaldehyde in PBS for 4 h, rinsed in PBS, dehydrated, wax-embedded and sectioned at 5 μm. Immunohistochemistry was performed on tissue sections using the following antibodies: ATMIN (1:1000, AB3271, Millipore), VANGL2 (1:500, gift from C. Dean; [42]), Inversin (1:500, ab65187, Abcam), β-catenin (1:500, 9582, Cell signalling), Fibrocystin (1:200, sc-99139, Santa Cruz), Caveolin-1 (3267, Cell Signalling). Alexa Anti-Rabbit 488 (1:500, ab150077, Abcam) or Alexa anti-rabbit 594 (1:500, ab150080, Abcam) was used as the secondary antibody.

### 2.10 Statistical methods

Data were analysed using unpaired two-tailed *t*-tests, unless otherwise stated. Significance was accepted at *P* < 0.05; error bars in all data represent standard error of the mean.

## 3 Results

### 3.1 The *Atmin*^*Gpg6*/*+*^ mouse line exhibits kidney abnormalities and mimics ARPKD

**Fig. 1:**
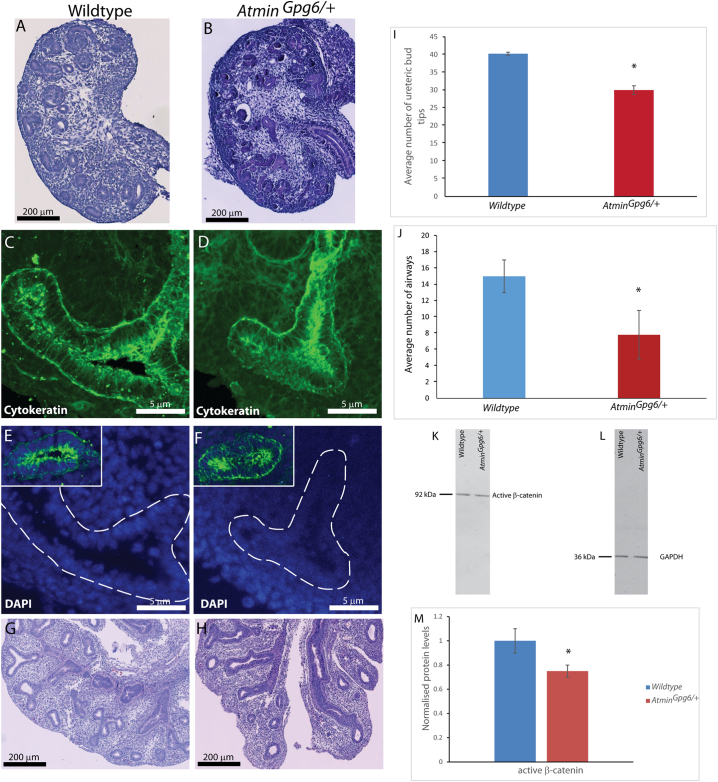
Cytoskeletal differences are detected in E13.5 *Atmin*^*Gpg6*/*+*^ embryos. *Atmin*^*Gpg6*/*+*^ kidneys (B) were smaller than wildtype (A), with a reduced number of ureteric bud tips (I). In z-stack images of wildtype (C, E) and *Atmin*^*Gpg6*/*+*^ (D, F) kidneys, epithelial cells appeared disorganised and were difficult to distinguish from the mesenchyme. *Atmin*^*Gpg6*/*+*^ lungs (H) were severely hypomorphic compared to age-matched wildtype lungs (G) and showed a reduction in the number of airways (J). Decreased active β-catenin expression was observed in *Atmin*^*Gpg6*/*+*^ kidneys compared to wildtype (K, L, M). Images are representative of at least six animals per group.

Our previous work on embryonic *Atmin*^*Gpg6*^ mice demonstrated that Atmin plays a role in kidney development by modulating Wnt signalling and provided evidence of severe lung hypoplasia in embryonic day (E) 13.5 *Atmin*^*Gpg6*/*Gpg6*^ mice [27,30]. As the *Atmin*^*Gpg6*/*Gpg6*^ homozygous mice display embryonic lethality and die around E14 [27], it was not possible to investigate whether the observed kidney defects would lead to renal cysts in post-natal mice. A detailed analysis of the *Atmin*^*Gpg6*/*+*^ mice, which survive to adulthood, was thus conducted. Histological examination of E13.5 wildtype and *Atmin*^*Gpg6*/*+*^ littermate kidneys stained with Periodic acid-Schiff (PAS) revealed a small reduction in size in the *Atmin*^*Gpg6*/*+*^ kidneys (Fig. 1A, B), accompanied by a reduced number of ureteric bud tips in the *Atmin*^*Gpg6*/*+*^ kidneys (40 vs 32, Fig. 1I), indicative of branching morphogenesis defects. After staining with pan-cytokeratin, E13.5 *Atmin*^*Gpg6*/*+*^ kidneys displayed shorter, broader ureteric bud tips compared to age-matched wildtype kidneys (Fig. 1D vs C), with epithelial cells appearing disorganised and randomly orientated, making them difficult to distinguish from the surrounding mesenchyme (Fig. 1E, F and insets). E13.5 *Atmin*^*Gpg6*/*+*^ mice also manifested lung hypoplasia (Fig. 1H) and a considerable reduction in the average number of airways (Fig. 4J), relative to the lungs of age-matched wildtype mice (15 vs 8, Fig. 1G, J).

**Fig. 2:**
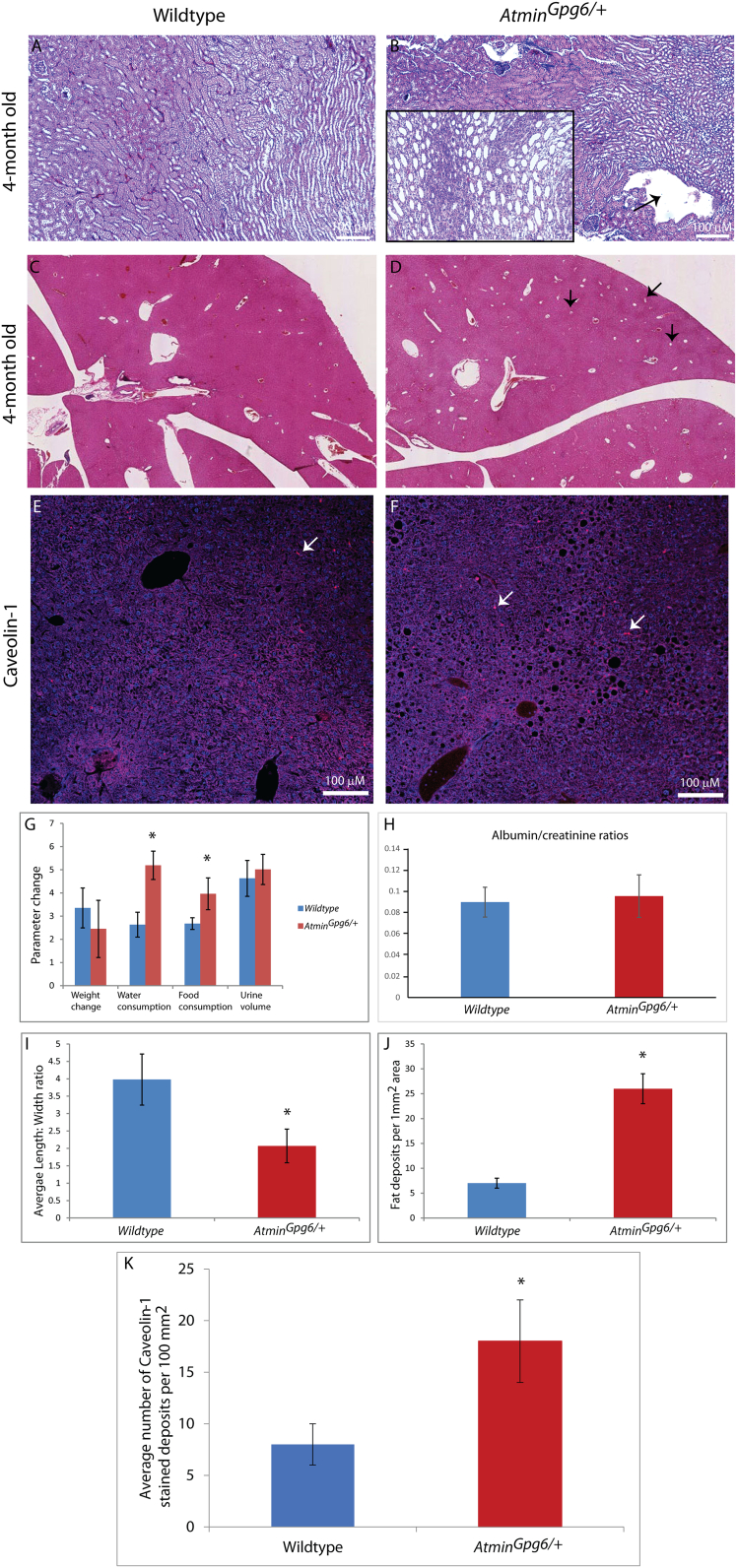
The *Atmin*^*Gpg6*^ phenotype mimics ARPKD. 4-month old *Atmin*^*Gpg6*/*+*^ kidneys stained with haemotoxylin and eosin showed tubule dilation (black box, B and I) and disorganised (black arrows) ureteric-bud derived structures (B) that were not observed in age-matched wildtype kidneys (A). Increased fat deposits were detected in *Atmin*^*Gpg6*/*+*^ livers (D, black arrows, J), a phenotype which was not seen in normal littermates (C; *n* = 8 per genotype). The increased number of fat deposits was confirmed by caveolin-1 staining of wildtype (white arrows, E) and *Atmin*^*Gpg6*/*+*^ (white arrows, F) livers, with an average of 8 vs 18 caveolin-1 stained deposits per 100 mm^2^ (K) being identified. Increased water and food consumption was observed in 4-month old *Atmin*^*Gpg6*/*+*^ mice (G, n = 12 per group, *p < 0.05) without a change in body weight or urine production or a significant difference in the albumin/creatinine ratio between *Atmin*^*Gpg6*/*+*^ and normal littermates (H).

**Supplementary Fig. S1:**
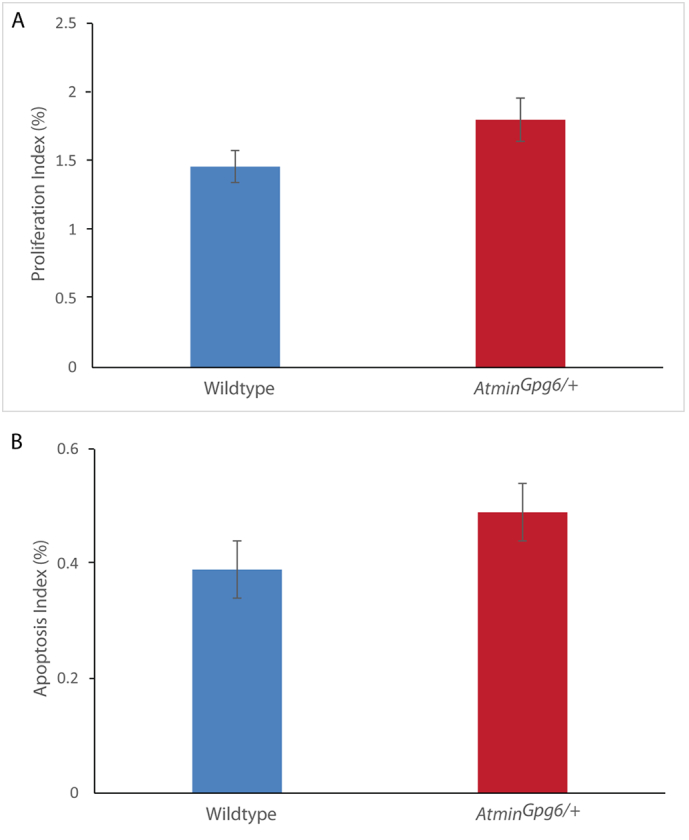
Proliferation and apoptosis are unaffected in E13.5 *Atmin*^*Gpg6*/*+*^ kidneys. Analysis of the percentage of proliferating cells in E13.5 whole kidney sections by immunostaining with anti-phospho histone H3 (A) or of apoptosis with anti-cleaved caspase 3 (B) revealed no significant differences in proliferation or apoptosis between *Atmin*^*Gpg6*/*+*^ and age-matched wildtype kidneys (n = 4 per genotype).

To investigate the underlying cause for the reduced number of epithelial structures in E13.5 *Atmin*^*Gpg6*/*+*^ kidneys, potential alterations in proliferation or apoptosis were investigated. No significant difference was detected in the percentage of proliferating cells (Supplementary Fig. S1A) or in the levels of apoptosis (Supplementary Fig. S1B) in *Atmin*^*Gpg6*/*+*^ kidneys compared to age-matched wildtype. When investigating β-catenin protein expression in E13.5 *Atmin*^*Gpg6*/*+*^ and age-matched wildtype kidneys, a mild but statistically significant 25% reduction in active β-catenin was observed in *Atmin*^*Gpg6*/*+*^ kidneys compared to age-matched, wildtype litter mates (Fig. 1K, L, M, p < 0.05).

**Supplementary Fig. S2:**
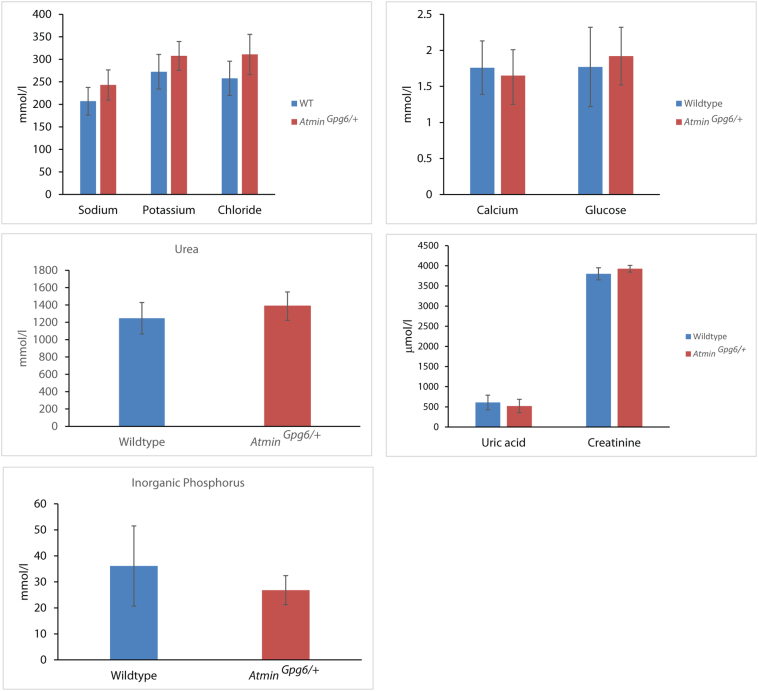
No significant differences in urine sodium, potassium, chloride, calcium, glucose, urea, uric acid, creatinine and inorganic phosphorus levels were detected in 4-month old *Atmin*^*Gpg6*/*+*^ mice compared to age-matched wildtype littermates.

An investigation for kidney abnormalities was also conducted in 4-month old *Atmin*^*Gpg6*/*+*^ mice compared to age-matched wildtype littermates. Interestingly, disorganised kidneys (black arrows, Fig. 2B) displaying dilated tubules (black box, Fig. 2B) were identified in *Atmin*^*Gpg6*/*+*^ mice (average length to width ratio of dilated tubules = 2.069, Fig. 2I), a phenotype that was not detected in age-matched wildtype kidneys (average length:width ratio of kidney tubules = 3.98, Fig. 2I). The *Atmin*^*Gpg6*/*+*^ mice manifested a statistically significant increase in food and water consumption, without a noticeable change in body weight or urine production (Fig. 2G), a change in the albumin/creatinine ratios (Fig. 2H) or significant differences in urine sodium, potassium, chloride, calcium, glucose, urea, uric acid, creatinine and inorganic phosphorus levels (Supplementary Fig. S2) compared to age-matched wildtype littermates. Furthermore, *Atmin*^*Gpg6*/*+*^ livers displayed an increased number of fat deposits (26 fat deposits/1mm^2^ liver area, black arrows, Fig. 2D, J), compared to normal age-matched livers (7 fat deposits/1mm^2^ liver area, Fig. 2C, J, p < 0.01). This was verified by caveolin-1 immunostaining that echoed the increase in fat deposits in the *Atmin*^*Gpg6*/+^ liver compared to age matched wildtype (18 fat deposits per 100 mm^2^ versus 8 fat deposits per 100 mm^2^, Fig. 2E, F, K). Thus it appears that the *Atmin*^*Gpg6*^ mouse line that shows combined defects in the kidney, liver and lung could provide a novel tool for the study of the molecular mechanisms of ARPKD.

### 3.2 *Atmin* modulates *Pkhd1* levels in mouse inner medullary collecting duct 3 cells

**Fig. 3:**
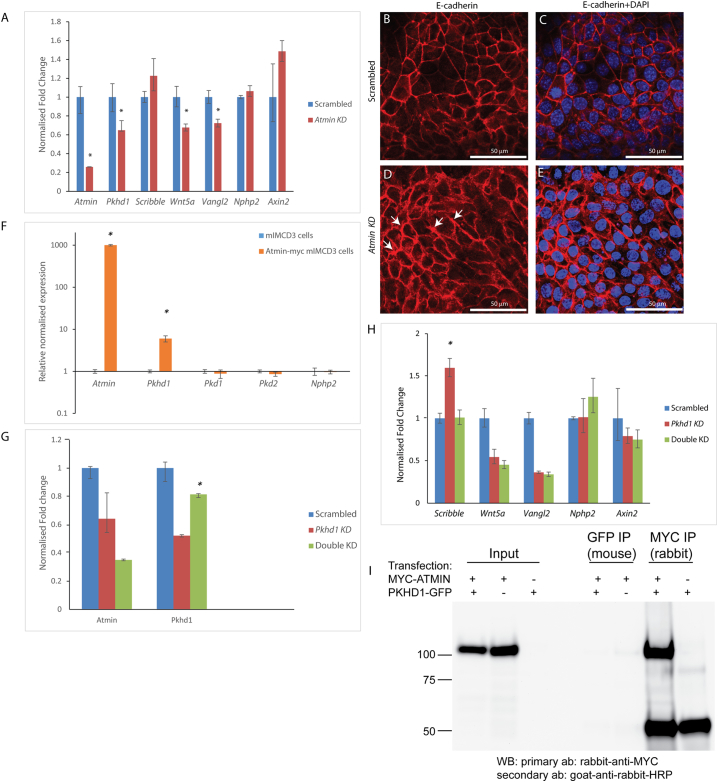
*Atmin* modulates *Pkhd1* mRNA expression in mIMCD3 cells. (A) A 0.35-fold decrease in *Pkhd1* mRNA was seen in the *Atmin* siRNA knockdown, accompanied by a 0.3-fold decrease in *Vangl2* and a 0.33-fold decrease in *Wnt5a* mRNA levels (n = 3, *p < 0.05) (B-E) Increased E-cadherin expression is observed in the *Atmin* knockdown cells compared to scrambled control. Substantially expanded E-cadherin distribution (white arrows) was only seen in the *Atmin* knockdown cells (n = 4 per experimental condition). (F) In the Atmin-myc transfected mIMCD3 cells, a 10^3^-fold increase in *Atmin* and a 6-fold increase in *Pkhd1* was detected (*n* = 3, *p < 0.05). (G) A 0.4-fold reduction in *Atmin* mRNA expression was observed in the *Pkhd1* siRNA knockdown (red). The double *Atmin*-*Pkhd1* siRNA knockdown (green) resulted in a 0.4-fold increase in *Pkhd1* mRNA expression, restoring *Pkhd1* expression to levels close to scrambled (blue, n = 3, *p < 0.05). (H) A 0.6-fold increase in *Scribble* mRNA expression was seen in the *Pkhd1* siRNA knockdown, whereas *Scribble* mRNA expression was restored to the levels detected in the scrambled control upon simultaneous *Atmin* siRNA knockdown. (n = 3,* *p* < 0.01). A 0.5-fold decrease in *Wnt5a* and *Vangl2* mRNA expression was revealed in the *Pkhd1* siRNA knockdown and this effect was not rescued by *Atmin* siRNA knockdown. No significant difference was detected in *Nphp2* and *Axin2* mRNA expression in any siRNA knockdown (n = 3, *p* > 0.05). For each experiment in A, F, G, H, at least three biological samples were used per group and for each sample, three technical replicates were performed. (I) Immunoprecipitation of ATMIN-MYC from lysates of HEK293T cells co-transfected with *Atmin*-*Myc* cDNA and C-terminal *Pkhd1*-*Gfp* cDNA and Western blot analysis did not reveal an interaction between the overexpressed proteins.

**Supplementary Fig. S3:**
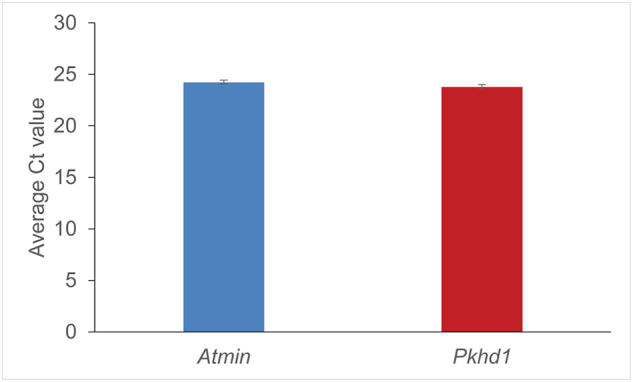
*Atmin* and *Pkhd1* mRNA expression in mIMCD3 cells. An average Ct value of 24.22 was observed for *Atmin* (blue), while *Pkhd1* (red) was detected at a Ct value of 23.76. n = 3, error bars represent standard error mean.

Many mutations associated with human disease result in a modulation of gene expression as opposed to its complete eradication. It is also informative to study transcriptional regulation by modifying levels of mRNA expression, as a clear readout of the relationship between genes. To investigate whether *Atmin* could affect *Pkhd1* transcription, siRNA knockdown experiments were conducted in mouse inner medullary collecting duct 3 (mIMCD3) cells that express high mRNA levels of *Atmin* and *Pkhd1* (Supplementary Fig. S3), with the aim of reducing but not completely abolishing gene expression, in order to be able to examine *Atmin*-*Pkhd1* interactions. Single *Atmin* knockdowns were performed in mIMCD3 cells, resulting in an 80% reduction in *Atmin* mRNA expression (Fig. 3A). Consistent with previously published work [30], *Atmin* knockdowns reduced *Vangl2* mRNA expression by 30% (Fig. 3A, red). In addition, a 33% decrease was observed in *Wnt5a* mRNA expression, while the *Scribble*, *Nphp2* (the gene that encodes Inversin) and *Axin2* mRNA levels remained unchanged. Furthermore, a notable increase in E-cadherin expression was seen in the *Atmin* knockdown cells (Fig. 3D, E) compared to scrambled controls (Fig. 3B, C), as well as markedly expanded areas of E-cadherin distribution being frequently observed only in the *Atmin* knockdowns (white arrows, Fig. 3D).

Interestingly, in the single *Atmin* knockdowns a statistically significant 35% decrease in *Pkhd1* mRNA levels was also observed (Fig. 3A). To further investigate the *Atmin*-*Pkhd1* relationship, full length myc-tagged Atmin was overexpressed in mIMCD3 cells. This caused a 6-fold increase in *Pkhd1* mRNA levels (Fig. 3F, orange), without affecting *Pkd1*, *Pkd2* or *Nphp2* mRNA expression.

Moreover, *Pkhd1* alone and *Pkhd1* together with *Atmin* were knocked down in mIMCD3 cells. *Pkhd1* knockdown resulted in a 50% decrease in *Pkhd1* expression (Fig. 3G, red); interestingly, this was accompanied by a 36% reduction in *Atmin* expression. *Atmin*-*Pkhd1* double knockdown resulted in a 66% decrease in *Atmin* levels. Surprisingly, this decrease in *Atmin* expression restored *Pkhd1* expression to levels similar to those of the scrambled control samples (81% *Pkhd1* expression in *Atmin*-*Pkhd1* double knockdown compared to 100% *Pkhd1* expression in scrambled).

Further investigation into the mRNA expression levels of non-canonical Wnt/PCP genes demonstrated a 45% decrease in *Wnt5a* and a 65% reduction in *Vangl2* mRNA expression in the *Pkhd1* knockdown cells (Fig. 3H, red). The additional reduction in *Atmin* expression in the Atmin-*Pkhd1* knockdown (Fig. 3H, green) did not significantly change *Wnt5a* and *Vangl2* expression. Moreover, the *Pkhd1* knockdown caused a 60% increase in *Scribble* levels, which was restored to normal expression levels in the *Atmin*-*Pkhd1* double knockdown (Fig. 3H), demonstrating that loss of *Atmin* can modulate *Scribble* mRNA expression. No change was observed in *Nphp2* and *Axin2*, either in the *Pkhd1* or the *Atmin*-*Pkhd1* double knockdowns.

**Supplementary Fig. S4:**
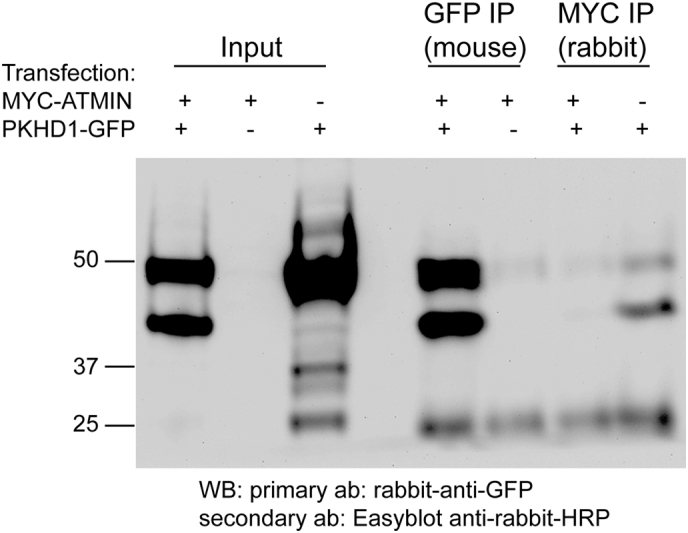
Immunoprecipitation using transfected HEK293T lysates. Immunoprecipitation of C-terminal Pkhd1-GFP from HEK293T cells transfected with both C-terminal *Pkhd1*-*Gfp* cDNA and *Atmin*-*Myc* cDNA did not reveal an interaction between the overexpressed proteins.

The C-terminus of Fibrocystin has been demonstrated to contain important protein binding motifs, such as a nuclear localisation signal [43], a ciliary target sequence [44] and a PC-2 binding domain [45], making it a likely candidate for protein-protein interactions. To shed further light on the interaction between *Atmin* and *Pkhd1*, full length myc-tagged Atmin and the last 193 amino acids of the C-terminus of GFP-tagged Fibrocystin were over-expressed in HEK293T cells. Both forward (Fig. 3I) and reverse (Supplementary Fig. S4) co-IPs did not show a direct interaction between Atmin and the C-terminus of Fibrocystin.

### 3.3 Atmin has no effect on apoptosis but reduces cellular proliferation and adhesion in cells lacking *Pkhd1*

**Fig. 4:**
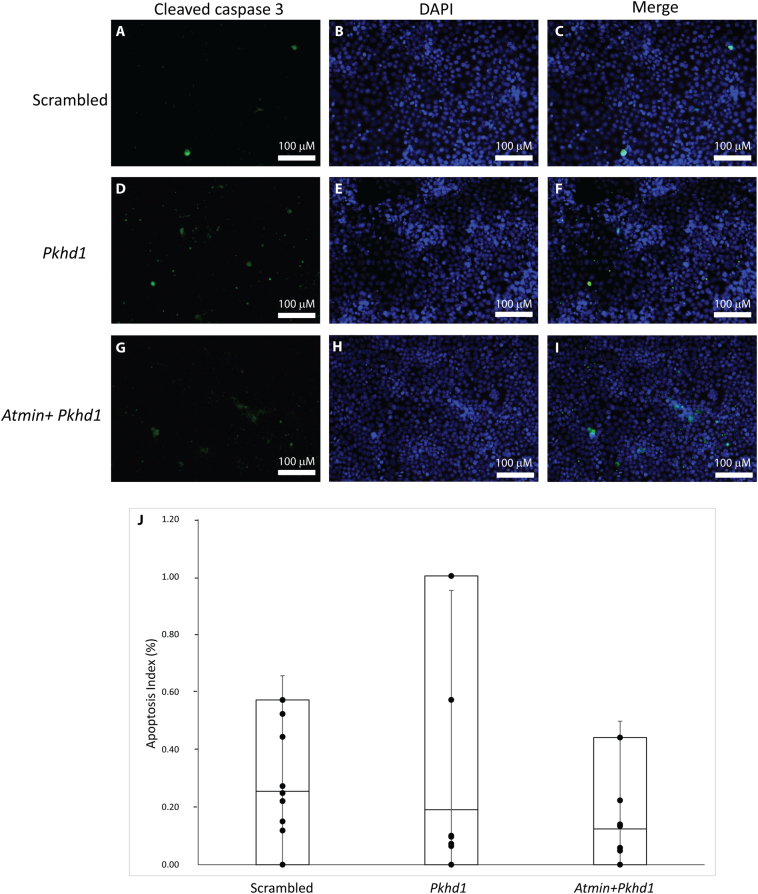
Apoptosis is unaffected by the *Pkhd1* and *Atmin*-*Pkhd1* siRNA knockdown in mIMCD3 cells. Quantitation of the percentage of apoptotic mIMCD3 cells stained with anti-cleaved caspase 3 (A, D, G) and DAPI (B, E, H) revealed no significant differences in apoptosis (J) between scrambled control (C), *Pkhd1* siRNA knockdown (F) and *Atmin*-*Pkhd1* siRNA knockdown (I, n = 3, p > 0.05).

**Fig. 5:**
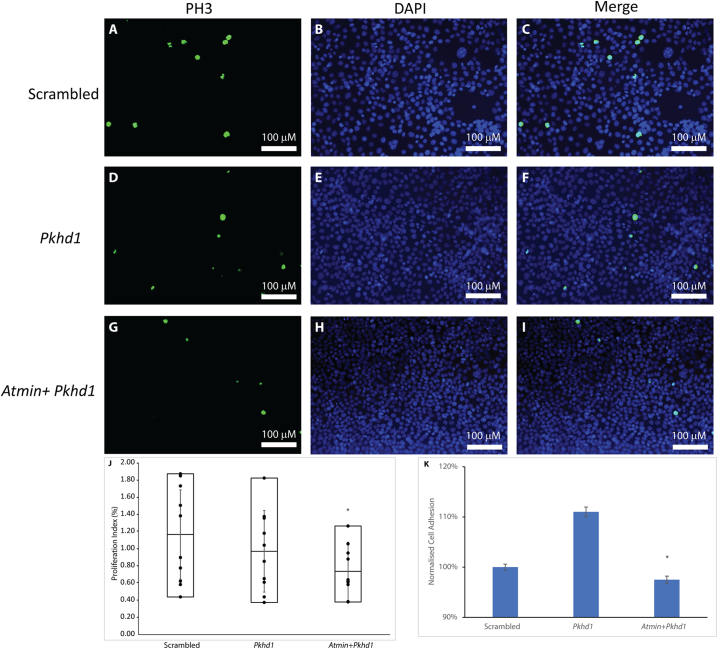
Loss of *Atmin* impacts cellular proliferation and adhesion in the *Atmin*-*Pkhd1* siRNA knockdown in mIMCD3 cells. Analysis of the percentage of proliferating mIMCD3 cells stained with anti-phospho histone H3 (A, D, G) and DAPI (B, E, H) revealed no significant difference in proliferation (J) between scrambled control (C) and *Pkhd1* siRNA knockdown (F, n = 3, p > 0.05). A statistically significant decrease in proliferation was observed in the *Atmin*-*Pkhd1* siRNA knockdown (I, J, n = 3, *p < 0.05). (K) Increased cellular adhesion was detected in *Pkhd1* siRNA knockdown but it was restored to scrambled control levels in the *Atmin*-*Pkhd1* siRNA knockdown (n = 3, *p < 0.05).

Since ARPKD has been associated with alterations in cellular proliferation and apoptosis [4,15], both were investigated in the *Pkhd1* and *Atmin*-*Pkhd1* knockdowns, where the transcripts had been reduced to ~50%. No changes in apoptotic indices were found between scrambled, *Pkhd1* and *Atmin*-*Pkhd1* knockdowns. The numbers of apoptotic cells, detected with cleaved caspase 3 antibody, were similar in scrambled (0.25%, Fig. 4A, C, J), *Pkhd1* (0.19%, Fig. 4D, F, J) and *Atmin*-*Pkhd1* (0.12%, Fig. 4G, I, J, *p* > 0.1) siRNA knockdowns. Furthermore, no significant difference in the number of proliferating cells stained with phospho-histone H3 (PH3) was observed between the scrambled control (Fig. 5A, C) and *Pkhd1* knockdowns (Fig. 5D, F) and the average proliferative indices were similar (1.1% for scrambled, 0.99% for *Pkhd1*, Fig. 5J). Importantly, a statistically significant decrease in the number of proliferating cells was seen in *Atmin*-*Pkhd1* double knockdown compared to the *Pkhd1* single knockdown (Fig. 5G, I), with an average proliferation index of 0.73% (Fig. 5J, *p* < 0.01). In addition, increased cell adhesion was observed in the *Pkhd1* knockdowns compared to scrambled controls (111% vs 100%, Fig. 5K). Significantly, in the *Atmin*-*Pkhd1* double knockdown, cell adhesion levels were restored to control (98%, Fig. 5 K).Thus, a reduction in *Atmin* expression in the context of *Pkhd1* knockdown can affect cellular proliferation and adhesion, signifying that changes in *ATMIN* expression may impact on cellular changes in ARPKD cystic epithelia.

### 3.4 A substantial increase in non-canonical Wnt/PCP signalling is detected in ARPKD

**Fig. 6:**
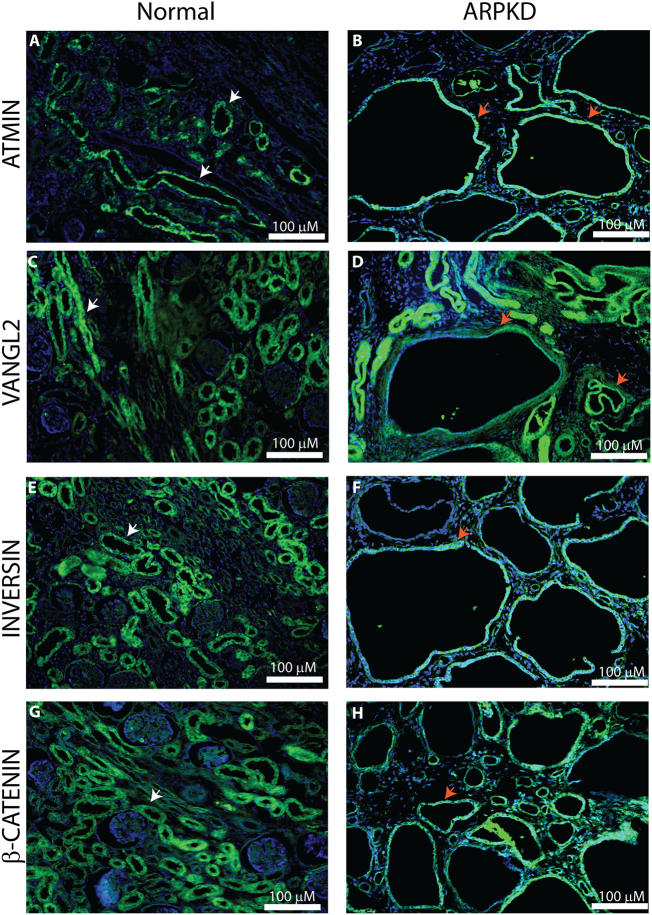
Immunohistochemistry of Wnt signalling proteins reveals differential localisation patterns between paediatric normal and ARPKD kidneys. ATMIN (A), VANGL2 (C), Inversin (E) and β-catenin (G) were expressed in ureteric bud-derived collecting tubules in normal human paediatric kidneys (white arrows). In age-matched ARPKD kidneys, strong ATMIN (B) and VANGL2 (D) expression was detected in cyst-lining epithelia (orange arrows). Moderate Inversin (F) and β-catenin (H) expression was also observed in ARPKD cyst-lining epithelia (red arrows; n = 4 per group).

**Supplementary Fig. S5:**
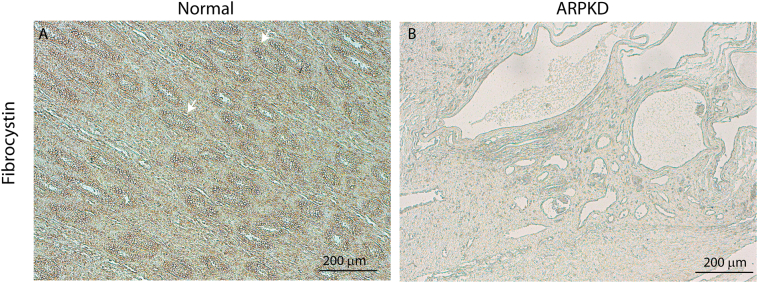
Fibrocystin expression in age-matched normal and ARPKD kidneys. Immunohistochemistry for Fibrocystin revealed strong expression in the tubules of paediatric kidneys (A, white arrows), which expression was absent in age-matched ARPKD kidneys (B, n = 4).

Altered cytoskeletal rearrangements may be caused by defective non-canonical Wnt/PCP signalling and abnormal Wnt signalling has been implicated in ADPKD [39]. Our published work showed that Atmin modulates non-canonical Wnt/PCP signalling [27] and since *Atmin* affected *Pkhd1* mRNA levels, the expression patterns of core and effector PCP proteins were examined in our previously characterised [18] normal (strong Fibrocystin expression) and ARPKD (no Fibrocystin expression, very large cysts) kidney tissues (Supplementary Fig. S5). In normal human paediatric kidneys, ATMIN, VANGL2, Inversin and β-catenin were expressed in ureteric bud epithelia (Fig. 6, white arrows). Apparently higher levels of ATMIN and VANGL2 were observed in ARPKD paediatric kidneys compared to age-matched normal kidneys, including strong expression in cyst-lining epithelia (Fig. 6, red arrows). Inversin and β-catenin were also highly expressed in ARPKD cyst-lining epithelia (Fig. 6, red arrows), although overall levels of renal expression appeared to be similar to those in normal kidneys. To investigate potential changes in gene expression of Wnt signalling components, a thorough analysis of mRNA expression levels of various canonical and non-canonical Wnt signalling genes was conducted.

**Fig. 7:**
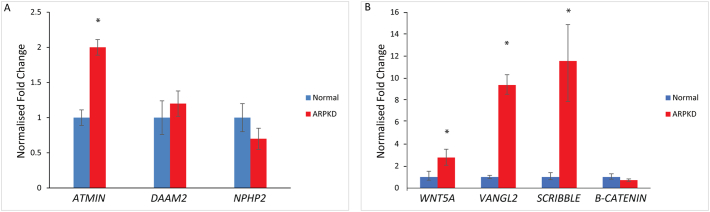
Increased non-canonical Wnt mRNA expression is detected in ARPKD tissues. (A) A statistically significant 2-fold increase in *ATMIN* mRNA expression was observed in ARPKD kidneys (red, *p < 0.01) compared to normalised, age-matched controls (blue). No significant difference in *DAAM2* and *NPHP2* expression was detected between ARPKD and normal kidneys (n = 11 per group, p > 0.05). (B) A 3-fold increase in *WNT5A* mRNA expression was observed in ARPKD kidneys (red, *p < 0.01) compared to age-matched normal kidneys (blue). A 9-fold increase in *VANGL2* and an 11-fold increase in *SCRIBBLE* expression was seen between ARPKD (red, *p < 0.01) and normal (blue) kidneys. No significant difference in *β*-*catenin* expression was detected between ARPKD (red) and normal (blue) kidneys (p > 0.05). *n* = 6 per group.

Across a number of ARPKD kidneys taken from patients who had reached end stage renal disease aged 0–18 years, a significant 2-fold increase in *ATMIN* mRNA expression was consistently observed compared to normal paediatric kidneys (Fig. 7A, *n* = 11, *p* < 0.01). No difference in *DAAM2* or *NPHP2* expression was detected between ARPKD and normal paediatric kidneys. In addition, in a severe ARPKD sub-group where patients reached end stage renal disease at an age of 5 years or younger, there was a 2.7-fold increase in *WNT5A* mRNA expression (Fig. 7B, *n* = 6, *p* < 0.05). In the same ARPKD kidneys, there was a 9-fold increase in *VANGL2* and an 11-fold increase in *SCRIBBLE* mRNA expression, compared to normal paediatric kidneys (Fig. 7B, n = 6, p < 0.01). Importantly, no change in *β*-*catenin* mRNA expression was seen between normal and ARPKD kidneys, demonstrating that ARPKD is associated with increased mRNA levels of non-canonical Wnt/PCP components.

**Fig. 8:**
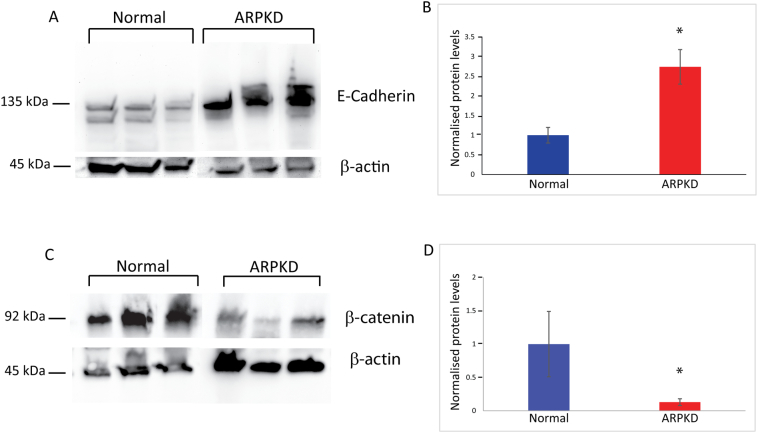
Altered E-cadherin and β-catenin expression in ARPKD kidneys compared to age-matched normal kidneys. Western blotting revealed a 2.74-fold increase (A, B) in E-cadherin (E) and a 0.87-fold decrease in β-catenin (C, D) protein levels in ARPKD compared to age-matched normal kidneys (n = 3, *p < 0.01). β-actin was used as a loading control.

Consistent with published work that showed that ARPKD cells exhibited increased adhesive properties and decreased polarised migration [18], a 2.74-fold increase in E-cadherin expression was observed in paediatric ARPKD kidneys compared to age-matched normal kidneys (Fig. 8A, B), signifying increased calcium-dependent cell-cell adhesion. Given that E-cadherin has been shown to interact with β-catenin [46], the expression of total β-catenin protein was assessed in normal and ARPKD kidneys. A striking 87% decrease in normalised total β-catenin levels was seen in ARPKD compared to age-matched normal kidneys (Fig. 8C, D), indicating that non-canonical Wnt/PCP signalling changes affect β-catenin protein but not mRNA levels in ARPKD.

## 4 Discussion

### 4.1 Non-canonical Wnt/PCP signalling in ARPKD

Non-canonical Wnt/PCP signalling is a complex pathway that has been studied in various systems and disease processes; downstream effects of this pathway involve cytoskeletal rearrangements and transcriptional activation [25]. Although the initial activation of the pathway (Wnt ligand binding to Fz receptor, Dvl attachment to the membrane) is similar to canonical Wnt signalling, the involvement of core PCP proteins such as the Vangls, results in variable outcomes that impact on cellular signalling processes. Our work has shown a role for non-canonical Wnt/PCP signalling in the autosomal recessive form of PKD for the first time. Mis-localisation and increased expression of core (VANGL2) and effector (ATMIN) PCP proteins were observed in ARPKD kidneys. Furthermore, increased expression of non-canonical Wnt genes (*WNT5A*, *VANGL2*, *ATMIN*, *SCRIBBLE*) was identified. The dramatic increase in *SCRIBBLE* associated with loss of Fibrocystin both in ARPKD kidneys and in *Pkhd1* siRNA knockdown cells demonstrates a potential involvement of Fibrocystin in Scribble regulation. This is complemented by a ~3-fold increase in E-cadherin that is most likely related to the known interaction of Scribble with E-cadherin [47] and the significant cytoskeletal re-organisation previously observed in ARPKD cystic epithelial cells [18]. It also opens up the possibility that there is a direct association between increased non-canonical Wnt/PCP signalling and end-stage renal disease observed in ARPKD patients.

In the kidney, Inversin has been considered to act as a switch between the canonical and non-canonical Wnt pathways [26]. Although a significant reduction in β-catenin protein (but not mRNA) expression was noted in ARPKD kidneys, the expression of *NPHP2* mRNA remained unchanged both in ARPKD kidneys and in siRNA knockdown experiments in mIMCD3 cells. This implies that *NPHP2* transcription is unlikely to have significant involvement in ARPKD and that the observed reduction in total β-catenin levels in ARPKD is a consequence of increased non-canonical Wnt/PCP signalling, as dys-regulation of the non-canonical Wnt/PCP pathway can impact on canonical Wnt signalling.

Thus loss of Fibrocystin results in mis-regulated non-canonical Wnt/PCP signalling, both in ARPKD kidneys and in the mIMCD3 mouse collecting duct cell line. The observed discrepancies in the *Wnt5a* and *Vangl2* mRNA levels between knockdown mouse collecting duct cells and human ARPKD kidneys might be due to either increased *ATMIN* levels in ARPKD kidneys, modulation of which significantly affects non-canonical Wnt/PCP signalling or differences in the transcriptional regulatory mechanisms of Fibrocystin between mouse and human. This is not surprising, given the different observed phenotypes of the *Pkhd1* knockout mice compared to ARPKD patients.

### 4.2 The Atmin-Fibrocystin relationship

The most significant relationship that our work has dissected is the one between ATMIN and Fibrocystin. Cytoskeletal, PCP-related defects were observed in embryonic *Atmin*^*Gpg6*/*+*^ mice and kidney and liver abnormalities were detected in adult *Atmin*^*Gpg6*/*+*^ mice. Modulation of *Atmin* levels in mIMCD3 cells affected *Pkhd1* mRNA expression and in the double knockdowns, a simultaneous reduction in *Atmin* and *Pkhd1* levels impacted cellular proliferation and adhesion. Interestingly, no direct interaction between Atmin and the C-terminus of Fibrocystin was observed by co-immunoprecipitation. Although the C-terminus of Fibrocystin is believed to contain its nuclear localisation signal and protein interaction domains, recent work has demonstrated that loss of the C-terminus does not significantly affect Fibrocystin function [48], raising questions as to its exact role. This could mean that Atmin may interact with Fibrocystin through other domains of the Fibrocystin protein or that there is genetic/intermediary protein interaction between Atmin and Fibrocystin that influences its transcriptional/translational regulation. In fact, STRING analysis has predicted that both ATMIN and Fibrocystin interact with serine-threonine kinases that might prove important for their function (data not shown). It is also possible that there is a role for epigenetics in the Atmin-Fibrocystin relationship and our future work will investigate for *Atmin* variants in paediatric and adult ARPKD, in order to be able to shed more light on the mechanisms of the Atmin-Fibrocystin interaction.

Although no change in proliferation indices was found when comparing all epithelial cells in wildtype and *Atmin*^*Gpg6*/*Gpg6*^ kidneys [30], statistically significant differences were seen in the proliferative indices of *Atmin*-*Pkhd1* double knockdowns compared to *Pkhd1* knockdowns and scrambled controls in mIMCD3 cells (Fig.5). It is hence possible that Atmin, at least in vitro, specifically affects the proliferation of cells of collecting duct origin, such as mIMCD3 cells, which were the focus of our current study. Nevertheless, loss of Fibrocystin is associated with increased *ATMIN* expression in ARPKD kidneys and consistent with our published work [30], loss of Atmin and *Pkhd1* does not lead to changes in apoptosis.

Recent work by Clark et al., 2018 has shown that ATMIN contains a flexible, disordered region that allows it to control target gene expression [49]. In the case of ATMIN's known downstream target, LC8 [27,50], ATMIN binding on LC8 increases LC8 transcription to produce more LC8. Once there is enough LC8, LC8 binds on ATMIN's disordered region and switches off LC8 transcription. As more LC8 binds on ATMIN, the rate of LC8 transcription decreases, this fine tuning being achieved by the number of ATMIN binding sites that are occupied by LC8. In addition, LC8 binding to the C-terminal of ATMIN alters its subcellular localisation [51], demonstrating that ATMIN's downstream targets can modify its function. This complex transcriptional regulatory role of ATMIN could apply to its other downstream targets, such as Fibrocystin. It should be noted that our data shows different outcomes in Atmin levels when there is Fibrocystin expression (knockdown experiments in IMCD3 cells, Fig. 3) compared to when there is no Fibrocystin expression (ARPKD tissues, Supplementary Fig. S5), implying that the levels of Fibrocystin might modulate ATMIN's transcription and levels of expression. Our future work will concentrate on dissecting how the levels of Fibrocystin may impact ATMIN's regulatory role in ARPKD.

Our work has also demonstrated that the *Atmin*^*Gpg6*^ mouse phenotype bears a similarity to paediatric ARPKD in humans, displaying kidney, liver and lung defects. As there is currently no single mouse model that displays the complete multi-organ spectrum of the ARPKD phenotype, our mouse line might provide a useful tool for the parallel study of all the tissues that are affected in severe, paediatric ARPKD, although there is still a need for additional mouse lines that model Caroli's disease more accurately. In conclusion, this work opens up the possibility that *Atmin* modulates *Pkhd1* and provides insights into novel signalling pathways that could impact ARPKD progression, raising exciting possibilities for future interventions and therapies.

The following are the supplementary data related to this article.

## Transparency document

Transparency document. Image 1
